# Immune Modulation and Immunotherapy in Solid Tumors: Mechanisms of Resistance and Potential Therapeutic Strategies

**DOI:** 10.3390/ijms26072923

**Published:** 2025-03-24

**Authors:** Suman Giri, Gopal Lamichhane, Jitendra Pandey, Ramesh Khadayat, Sindhu K. C., Hari Prasad Devkota, Dipendra Khadka

**Affiliations:** 1 Asian College for Advance Studies, Purbanchal University, Satdobato, Lalitpur 44700, Nepal; girisymo44@gmail.com; 2 Department of Nutritional Sciences, Oklahoma State University, Stillwater, OK 74078, USA; gopal.lamichhane@okstate.edu; 3 Department of Chemistry, University of Hawai’i at Manoa, 2545 McCarthy Mall, Honolulu, HI 96822, USA; jitendra@hawaii.edu; 4 Patan Hospital, Patan Academic of Health Sciences, Lagankhel, Lalitpur 44700, Nepal; rameshkhadayat123@gmail.com; 5 Department of Pharmacology, Chitwan Medical College, Tribhuwan University, Bharatpur-05, Chitwan 44200, Nepal; sindhukc119@gmail.com; 6 Graduate School of Pharmaceutical Sciences, Kumamoto University, Oehonmachi 5-1, Chuo-ku, Kumamoto 862-0973, Japan; devkotah@kumamoto-u.ac.jp; 7 Headquarters for Admissions and Education, Kumamoto University, Kurokami, 2-39-1, Chuo-ku, Kumamoto 860-8555, Japan; 8 NADIANBIO Co., Ltd., Wonkwang University School of Medicine, Business Incubation Center R201-1, Iksan 54538, Jeonbuk, Republic of Korea; 9 KHAS Health Pvt. Ltd., Dhangadhi-04, Kailali 10910, Nepal

**Keywords:** immune cells, tumor microenvironment, immune modulation, immunotherapy, therapeutic resistance, emerging strategies, therapeutic outcomes

## Abstract

Understanding the modulation of specific immune cells within the tumor microenvironment (TME) offers new hope in cancer treatments, especially in cancer immunotherapies. In recent years, immune modulation and resistance to immunotherapy have become critical challenges in cancer treatments. However, novel strategies for immune modulation have emerged as promising approaches for oncology due to the vital roles of the immunomodulators in regulating tumor progression and metastasis and modulating immunological responses to standard of care in cancer treatments. With the progress in immuno-oncology, a growing number of novel immunomodulators and mechanisms are being uncovered, offering the potential for enhanced clinical immunotherapy in the near future. Thus, gaining a comprehensive understanding of the broader context is essential. Herein, we particularly summarize the paradoxical role of tumor-related immune cells, focusing on how targeted immune cells and their actions are modulated by immunotherapies to overcome immunotherapeutic resistance in tumor cells. We also highlight the molecular mechanisms employed by tumors to evade the long-term effects of immunotherapeutic agents, rendering them ineffective.

## 1. Introduction

Over the past few decades, clinical successes have demonstrated a revolutionary shift in cancer therapies, moving away from chemotherapeutic agents that broadly target solid tumors to the utilization of cancer immunotherapies that actively modulate immune responses against cancer cells and the tumor immune environment. The breakthroughs in cancer immunotherapies have brought a new hope in the combat against cancer treatment in recent times [1]. Modulating specific immune-inflammatory cells within the tumor microenvironment (TME) represents a highly promising strategy, particularly in the context of cancer immunotherapy. The foremost initiation of cancer immunotherapy, known as immune checkpoint inhibitors (ICIs), including cytotoxic T lymphocyte antigen-4 (CTLA-4) and programmed cell death-1 (PD-1) or its major ligand PD-L1, is associated with increasing T cell functions against tumor cells [2]. In addition to ICIs, immunotherapies comprise adoptive cell transfer (ACT) (like chimeric antigen receptor T-cell (CAR-T) treatment), cancer vaccine, and oncolytic virotherapy (OVT), aiming to overcome immunotherapeutic resistance by targeting and modulating the immune cells and their functions [1,2]. Immunomodulatory drugs play a pivotal role in regulating tumor initiation, progression, metastasis, and the immune responses to standard cancer treatments [3]. Consequently, cancer immunotherapies represent a promising treatment strategy, interfering with specific proteins that control and eliminate how tumor cells grow, divide, and spread by influencing and controlling the immune system to recognize and eradicate cancer cells.

Recently, immune modulation and immunotherapy resistance have emerged as critical issues in oncology. Notably, ICIs exhibit a clinical response rate below 30%, primarily due to therapeutic resistance issues within the immune system. Solid tumors possess a heterogeneous and intricate genetic system that enables tumor cells to promote resistance to immunotherapies. For instance, tumor cells enhance the expression of PD-L1, inhibiting the activity of cytotoxic T lymphocytes (CTLs) by interacting with PD-1 on the surface of T cells [4]. At present, due to the heterogeneous environment of the tumor, immunotherapy resistance—whether intrinsic or acquired—remains a massive challenge that limits its universal effectiveness, prompting the exploration of effective combination therapy strategies to overcome it. Despite its transformative role in cancer treatment, immunotherapy faces several challenges that limit its universal effectiveness. Tumor heterogeneity enables cancer cells to evade immune detection, while the immunosuppressive TME recruits regulatory cells and cytokines that inhibit immune responses [5,6]. Adaptive resistance mechanisms, such as upregulating alternative immune checkpoints (LAG-3, TIM-3) and altering antigen presentation, further reduce therapeutic efficacy [7]. Low tumor mutational burden (TMB) results in fewer neoantigens, weakening immune recognition, while prolonged antigen exposure leads to T cell exhaustion and dysfunction [8,9]. Additionally, immune-related adverse events (irAEs) can cause severe toxicities in multiple organs, requiring immunosuppressive interventions that compromise treatment benefits [10]. The lack of reliable predictive biomarkers further complicates patient selection, highlighting the urgent need for novel strategies to overcome resistance and improve immunotherapy outcomes [11]. Nonetheless, various mechanisms associated with resistance to immunotherapy have been explored to improve the effectiveness of tumor immunotherapy and expand its clinical applications [12]. In this review, we concentrate on presenting a concise overview of major immune cells in the TME and their functional roles. We also explored various mechanisms contributing to immunotherapeutic resistance, emphasizing how modulating specific immune cells with immune modulators can enhance efficacy and provide sustained antitumor responses, showing potential for success in cancer immunotherapy. Furthermore, we have highlighted advancements in new delivery platforms designed to overcome immunotherapeutic resistance in cancer treatments.

## 2. Immune Modulation: The Most Potent Therapeutic Modality in Immuno-Oncology

**Figure 1:**
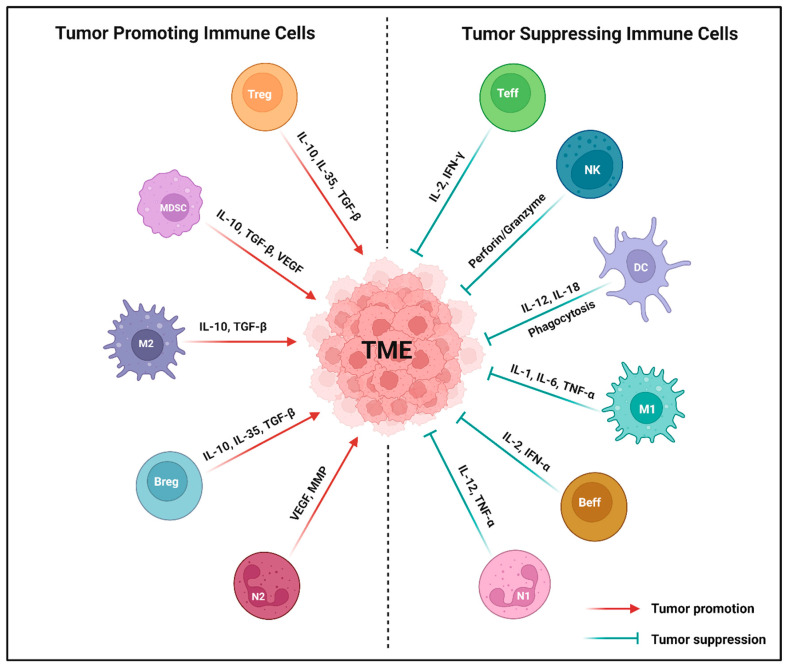
Major types of tumor-associated immune cells in the tumor microenvironment (TME). Both tumor-promoting and tumor-suppressing immune cells directly impact the phenotypes and functions in the TME through the release of various cytokines or chemokines. Tumor-promoting immune cells, such as myeloid derived suppressor cell (MDSC), N2-tumor associated neutrophil (N2), regulatory T cell (Treg), M2-tumor associated macrophage (M2), and regulatory B cell (Breg) significantly promote the TME by secreting IL-10, VEGF, TGF-β, MMPs, and IL-35. Conversely, tumor-suppressing immune cells like natural killer cell (NK), N1-tumor associated neutrophil (N1), effector T cell (Teff), M1-tumor associated macrophage (M1), dendritic cell (DC), and effector B cell (Beff) produce perforin or granzymes, IL-12, TNF-α, IL-2, IFN-γ, IL-1, IL-6, and IL-18 that suppress the TME. Created using BioRender.com.

Immune modulation is a powerful therapeutic strategy in modern cancer therapy, utilizing the immune system to target and eliminate malignant cells. An enhanced understanding of tumor–immune cell interactions within the TME has facilitated the application of strategies like ICIs, ACT, cytokine therapy, cancer vaccines, and OVT [13]. The cellular crosstalk between immune cells and the solid TME in cancer plays a vital role in both promoting and suppressing tumor progression. The presence and functions of specific immune cells are intricately regulated by various biological factors within the TME [14,15]. Based on their functional roles in tumor progression, immune cells can be broadly categorized into tumor-promoting and tumor-suppressing immune cells (refer to Figure 1 and Table 1).

### 2.1. Tumor-Promoting Immune Cells

**Tregs**, a well-recognized subset of CD4^+^ T cells, exert immunosuppressive control over immune response, regulating homeostasis and immune activation [16]. Their inhibitory effects on immune function involve diverse mechanisms, such as the release of immunomodulatory cytokines like IL-10, IL-35, and TGF-β; the cytolysis of effector immune cells through the secretion of perforin and granzyme molecules; and metabolic interference by cyclic adenosine monophosphate (cAMP)-mediated immunosuppression [17]. Moreover, Tregs produce and accumulate substantial levels of cAMP, which is then transported to target cells through intercellular communication via gap junctions. Tregs express FOXP3 as a critical transcription factor for their differentiation and regulatory function. This includes the secretion of suppressive cytokines (IL-10, IL-35, and TGF-β) and the expression of the inhibitory surface molecule (CTLA-4), which suppress the production of IL-2 and IFN-γ [18]. Found in high density within various solid tumors, Tregs exhibit significant diversity among CD4^+^ T cells in tumor-infiltrating lymphocytes (TILs) [19]. A high ratio of FOXP3^+^ Tregs to CD8^+^ T cells correlates with a poor prognosis in patients with breast, lung, gastric, and ovarian cancer [20]. Consequently, the presence of Tregs in the TME plays a pivotal role in shaping the antitumor immune response.

**MDSCs**, a diverse group of immature myeloid cells, play a key role in immune suppression associated with tumors. A key characteristic of MDSCs is their ability to suppress immune cell function, achieved through the expression of immune-regulating factors such as IL-10, TGF-β, inducible nitric oxide synthase (iNOS), cyclooxygenase-2 (COX-2), arginase-1 (ARG-1), and indoleamine 2,3-dioxygenase chelating cysteine (IDOCC) [14,21]. Within the TME, MDSCs undergo enhancement and proliferation in response to pro-inflammatory cytokines and chemokines released by tumor cells [22]. Apart from contributing to the immunosuppressive TME, MDSCs foster tumor development through non-immunological mechanisms [23] that promote de novo angiogenesis by releasing growth factors like vascular endothelial growth factor (VEGF), platelet derived growth factor (PDGF), and basic fibroblast growth factor (bFGF), altering the extracellular environment through matrix metalloproteinases (MMPs). Additionally, MDSCs contribute to tumor angiogenesis by upregulating IL-10 expression and reducing IL-12 expression [24]. Particularly, MDSCs induce MMP9 expression, leading to the degradation of the basement membrane and ECM, facilitating tumor cell entry into the bloodstream for migration to the metastatic site [25]. Within the TME, MDSCs use the STAT/MyD88 signaling pathway to deprive T cells from essential amino acids, activating metabolic enzymes like ARG1 and iNOS [26]. MDSCs are commonly recognized as regulators of Tregs in various solid tumors, with evidence indicating that Tregs-derived TGF-β plays a critical role in regulating the generation and function of MDSCs. This interaction presents promising immunotherapeutic strategies to promote T cell-mediated anticancer immunity [22].

**M2-TAMs**, characterized by an anti-inflammatory polarization, are immune cells within the TME that contribute to tumor promotion by inducing hypoxia and ECM accumulation, resulting in immunosuppression and sustained vascular normalization [14]. Despite their tumor-promoting role, M2-TAMs play a pivotal role in creating essential penetration channels for immune cells through vascular normalization. These cells exhibit anti-pro-inflammatory responses by promoting the release of IL-10 while reducing the production of IL-12 and IL-23 [27]. Additionally, M2-TAMs contribute to anti-inflammatory effects and support tissue repair [28].

**Bregs** represent another category of immune cells that contribute to tumor promotion. They engage in pro-tumorigenic activities by interacting with MDSCs, secreting suppressive cytokines (IL-10, IL-35, and TGF-β), and stimulating immunosuppressive Tregs [29]. Bregs have the capacity to eliminate macrophages, DCs, and other immune cells during tumor progression [30]. A recent study using bulk RNA sequencing has differentiated Bregs in tumors of responders and non-responders to ICIs, highlighting Bregs as potential therapeutic targets [31].

**N2-TANs** represent a tumor-promoting N2 phenotype within the TME, exhibiting potent immunosuppressive and tumor-promoting actions. These include the stimulation of tumor angiogenesis, invasion, and metastases through the synthesis and secretion of various biological factors like VEGFs, hepatocyte growth factors (HGFs), reactive oxygen species (ROS) or reactive nitrogen species (RNS), oncostatin M, neutrophils elastases (NEs), and MMPs [32]. Consequently, N2-TANs impact other immune cells in the TME, inhibiting T and NK cells responsible for killing tumor cells, and interactions with TAMs, ultimately promoting tumor progression and metastasis.

### 2.2. Tumor-Suppressing Immune Cells

**Teffs** are composed of cytotoxic CD8^+^ T cells and effector CD4^+^ T cells, which play a crucial role in suppressing tumors within the TME. Typically, cytotoxic CD8^+^ T cells interact with MHC-I molecules on APCs, differentiating into cytotoxic T lymphocytes that exhibit cytotoxicity against cancer cells [33]. While cytotoxic CD8^+^ T cells act locally at the tumor site, memory CD8^+^ T cells, functioning as resident memory T cells, either infiltrate the tumor or circulate in the bloodstream, serving diverse roles as central memory T cells [34]. These effector T cells release IFN-γ, inhibiting tumorigenic cells and tumor angiogenesis by suppressing EC proliferation and increasing the expression of cytokine-encoding genes like CXCL9, CXCL10, and CXCL11. These cytokines facilitate pericyte recruitment and immune cell migration across blood vessel walls [35]. Effector CD4^+^ T cells play a pivotal role in the antitumor immune response. They release IL-2, directly activating the cytotoxic CD8^+^ T cells expressing the IL-2 receptor subunit alpha or CD25 [36]. Moreover, effector CD4^+^ T cells indirectly stimulate DC activation, triggering cytotoxic CD8^+^ T cells through cross-presenting tumor antigens or generating effector cytokines like TNF-α and IFN-γ [37]. These cytokines directly exhibit antitumor activity by polarizing and activating T cells into T helper 1 phenotype (T_H_1). The reduction in CD4^+^ T_H_1 cells in various mouse tumor models is associated with decreased pericyte coverage and the development of malformed tumor blood vessels, while the activation of effector CD4^+^ T cells improves vessel normalization [35]. Within the TME, T_H_1 cells further polarize M2-TAMs into M1-TAMs, enhancing DC maturation and suppressing tumor angiogenesis [38]. Clinical evidence emphasizes the significance of effector CD4^+^ T cells in successful antitumor immune response. Single-cell RNA sequencing of T cells from biopsies of patients with colorectal carcinoma (CRC) suggested the favorable enrichment of T_H_1-like CD4^+^ T cells expressing effector cytokines like IFN-γ, CXCR5, and BHLHE40 as a transcription factor [39]. In the peripheral blood circulation, the presence of T_H_1-polarized CD4^+^ T cells is considered a positive prognosis in patients with CRC and non-small cell lung carcinoma (NSCLC) [40,41]. Tumor-infiltrating effector T cells, particularly those releasing IFN-γ, significantly contribute to vascular and immune remodeling.

**NK cells** serve as a critical first line of defense against solid tumors, constituting the cytotoxic compartment of the innate lymphoid cells during tumor progression [42]. Generally characterized as CD3^−^ CD56^+^ cells in humans, NK cells comprise approximately 5–15% of circulating lymphocytes in peripheral blood [43]. As proposed by Velichinskii et. al., NK cell effector function in the TME hinges on the balance between activating cytotoxicity receptors (NKp46, NKp44, NKp30, and NKG2D) and killer inhibitory receptors (NKG2A and KLRG1) [44]. NK cells are attracted to tumor cells by cytokines or chemokines released by DCs [45], influencing tumor-promoting or killing responses by secreting perforin and granzymes, enhancing target cell apoptosis. Furthermore, NK cells release the pro-inflammatory cytokines (IL-6, TNF-α, and IFN-γ) and chemokines (GM-CSF and CCL5), demonstrating direct antitumor efficacy and promoting innate and adaptive responses [43]. Serving as both cytotoxic and immunoregulatory cells, NK cells influence antitumor responses by regulating DC and T cell reactions. Despite their critical role in tumor immunosurveillance, a previous study suggests that the killing function of tumor-infiltrating NK cells is consistently suppressed [43]. For instance, NK cells in the TME of breast cancer exhibit an undeveloped phenotype with a reduced expression of DX5, CD27^low^CD11b^low^ phenotype, induction of NKG2A expression, low levels of NKp46, perforin, and granzyme B expression. Treatment with IL-12 and anti-TGF-β induces the maturation of tumor-infiltrating NK cells [46]. Cellular signaling through ICIs (PD-1, CTLA-4, CD96, TIM-3, TIGIT, or NKG2A) controls NK cell function, and blocking inhibitory pathways can restore NK cell-mediated antitumor immunity [43].

**DCs**, serving as potent professional APCs, initiate T cell activation and facilitate interactions with B cells and NK cells. DCs play a crucial role in immunological processes, influencing the establishment, modulation, and maintenance of immune responses [47]. Categorized as mature and immature, DCs originate from bone marrow myeloid progenitors. Mature DCs, including conventional DCs (cDCs) and plasmacytoid DCs (pDCs), express low levels of antigen uptake and induce T cells activation; however, immature DCs exhibit high antigen uptake but are less effective in T cell activation [48]. Notably, mature cDCs, especially cDC1, release anti-angiogenic cytokines (IL-12 and IL-18) and chemokines (CXCL9, CXCL10, and CCL21), suppressing cancer angiogenesis [48]. Distinguished by basic leucine zipper transcriptional factor ATF-3 (BATF3) and interferon regulatory factor 8 (IRF8), DCs play a pivotal role in triggering CD8^+^ T cell-mediated immunity [49]. BATF3 influences antitumor responses, impacting immunotherapies like ICIs and ACT, promoting CD8^+^ T cell infiltration into tumors [50]. Conversely, pDCs release IFN-α, suppressing EC proliferation and inducing anti-angiogenic cytokines and chemokines via TLR7 signaling [51]. DC-produced pro-inflammatory cytokines can enhance Treg proliferation and activation in the TME, leading to immune suppression and downregulation of co-stimulatory molecules on DCs [52]. Despite these challenges, ongoing research explores strategies like DC-based vaccines and combination therapies with ICIs to harness DC potential for cancer immunotherapy, aiming to enhance antitumor immune responses in patients [53].

**M1-TAMs**, a key component of myeloid cells in the TME, play a significant role in influencing tumor initiation, progression, and metastasis. TAMs can transition from M1 to M2 phenotype, promoting tumor angiogenesis by releasing the angiogenic CXC chemokines (CXCL8/IL-8 and CXCL12), pro-angiogenic growth factors (VEGF, EGF, and PDGF-β), and angiogenesis-associated factors (TGF-β) [54]. M1-TAMs exhibit pro-inflammatory immune responses and suppress tumor cell proliferation via releasing pro-inflammatory cytokines such as IL-1, IL-6, IL-12, IL-18, and TNF-α [54], attracting unpolarized M1-TAMs. Additionally, M1-TAMs release ROS intermediates or NO to enhance host defense and induce tumor cell-killing activity. In the early stage of tumorigenesis, M1-TAMs also suppress tumor angiogenesis, promote vessel maturation by releasing the anti-angiogenic cytokines (IL-12 and TNF-α), and activate antitumor immunity [14,54]. Correspondingly, IL-12-based cancer immunotherapy has been shown to reduce microvessel thickness and enhance M1-TAM polarization in various cancer types [55].

**Beffs** are a topic of controversy among tumor-suppressing immune cells, engaging in antitumor activities by directly eliminating tumor cells through Fas/FasL pathways. Therefore, Beffs exhibit a targeted and Fas ligand-dependent manner of killing of tumor cells [56]. They also impact DC movement, producing antibodies that prompt M1-TAMs to undergo antibody-dependent cellular cytotoxicity or antibody-dependent cellular phagocytosis for tumor cell elimination [57]. Cytokines can activate or induce Beffs, enhancing the B cell-mediated antitumor immune response. For example, IL-2 enhances the antitumor immune response of adoptively transferred tumor-draining lymph node (TDLN) B cells, while IL-17A improves B cell activity in esophageal squamous cancer [58]. Therefore, cytokine-related cancer therapies present a potential avenue to enhance Beffs and improve the antitumor efficacy of B cell-involved cancer immunotherapy.

**N1-TANs** exhibit potent antitumor activity through mechanisms such as direct or antibody-dependent cytotoxicity, ROS-mediated coupling, and the generation of neutrophil extracellular traps [59]. This activity is attributed to their production of pro-inflammatory or immune-stimulatory cytokines like IL-12, TNF-α, CXCL9, and CXCL10, which promote the activation of CD8^+^ T cells [60]. N1-TANs inhibit tumor progression via the activation of other immune cells such as NK cells, DCs, and T and B lymphocytes. Numerous studies have reported the significant contribution of N1-TANs in inhibiting tumor growth through various mechanisms, including direct cytotoxicity, induction of apoptosis in tumor cells, suppression of tumor growth, metastasis, and improvement of antitumor immune responses [61,62].

### 2.3. Contribution of Immunomodulators to the Antitumor Response

**Figure 2:**
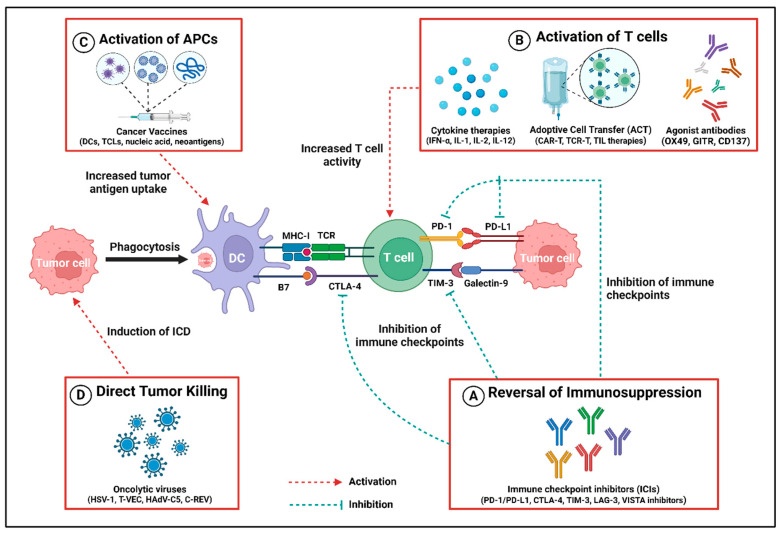
Different contributions to antitumor immune response by cancer immunotherapies. (**A**) Immune checkpoint inhibitors (ICIs) reverse the immunosuppression mediated by the immune checkpoints, e.g., PD-1/PD-L1, CTLA-4, TIM-3, LAG-3, and VISTA, by selectively inhibiting their receptors. (**B**) Immunomodulators such as cytokines (IFN-α, IL-1, IL-2, IL-12), adoptive cell transfer (ACT) (mainly CART-T, TCR-T, TIL therapies), and agonist antibodies (OX49, GITR, CD137) stimulate the activation and proliferation of T cells, thereby exerting the antitumor activity. (**C**) Cancer vaccines based on dendritic cells (DCs), tumor cell lysates (TCLs), nucleic acid, and neoantigens cause an increased uptake of tumor antigens, which results in the activation of antigen presentation cells (APCs) like DCs that directly activate the T cell to kill the tumor cell. (**D**) Virotherapy by utilizing oncolytic viruses (HSV-1, T-VEC, HadV-C5, C-REV) enhances antitumor response by inducing immunogenic cell death (ICD), resulting in a cascade of events leading to tumor cell clearance. Created using BioRender.com.

With a deeper understanding of the immune system’s influence on tumor outcomes, immunomodulators have emerged as powerful allies in combating aggressive malignancies. Cancer immunomodulators, also known as immunotherapy for cancer treatments, modulate various biological factors of the immune system, enhancing the identification and elimination of tumor cells [1,63]. Therefore, the contributions of immunotherapy impact not only the tumor cells but also the surrounding TME, leading to alterations in the composition of major cell types and their functions for antitumor immune response, as shown in Figure 2 and Table 2.

**ICIs** represent a revolutionary class of immune modulators in cancer immunotherapy, playing a pivotal role in regulating immune responses. They are key regulatory proteins expressed on T cells, and help to maintain immune tolerance by preventing autoimmunity and modulating immune responses to self-antigens. ICIs also counteract tumor-mediated suppression of antitumor immune responses, contrasting with strategies directly harming tumor cells [64]. The two prominent ICI approaches involve a CTLA-4 blockade and disruption of the PD-1/PD-L1 interaction, which play a pivotal role in immune response modulation and their dysregulated expression in the TME, hindering an effective elimination of tumor cells by Teffs [65]. CTLA-4 negatively regulates T cell responses, and anti-CTLA-4 therapy has shown promise in melanoma treatment by blocking inhibitory signals between APCs and T cells [66]. PD-L1 expression in endothelial cells downregulates CD8^+^ T cell activation and cytolysis, potentially enhancing Tregs activation and cytokine secretion [67]. Additionally, emerging immune checkpoints like LAG-3, TIM-3, VISTA, and TIGIT are potential targets for T cell cancer immunotherapy. LAG-3 modulates lymphocyte–tumor cell interactions, directing T cell suppression [68]. TIM-3 plays a crucial role in tumor-associated immune response and combining TIM-3 and PD-1 inhibitors enhances tumor regression and induces antitumor T cell responses [69]. TIM-3 interaction with Galectin-9 suppresses innate and adaptive immunity, leading to T cell and NK cell exhaustion [70]. VISTA-based ICI therapies reduce suppressive effects on T cells by interacting with MDSCs [71], and anti-TIGIT therapies promote T and NK cell functions, boosting multiple antitumor immunity mechanisms across various solid tumor types [72]. Despite the remarkable clinical efficacy of ICIs, only a subset of patients responds to the therapy, with certain tumor histologies remaining entirely refractory. Resistance to ICIs is more frequently observed in immune-excluded and immunologically “cold” tumors [73]. Moreover, irAEs pose another significant challenge in ICI therapy, which affects multiple organs, namely skin, musculoskeletal system, gastrointestinal tract, cardiovascular system, and endocrine system [74,75]. These reactions range from chronic conditions to severe life-threatening complications such as cytokine storm [73,76]. Therefore, the development of reliable predictive biomarkers for response, resistance, and toxicity is crucial to improve patient selection and maximize the effectiveness of ICI therapy.

**Cytokines**, serving as immunomodulators, marked the inception of cancer immunotherapy with the approval of interferons as recombinant IFN-α therapies. In recent years, the primary cytokines explored for immunotherapy include interferons, interleukins, and GM-CSF. IFN-α is produced by immune cells in response to pathogens, and enhances the activation and maturation of lymphocytes, macrophages, DCs, and NK cells, with interferon-based therapies inhibiting tumor angiogenesis in the extracellular tumor area [77]. Interleukins (IL-1, IL-2, or IL-12) stimulate CD4^+^ T cell, CD8^+^ T cell, and B cell activation and differentiation, promoting innate and adaptive immune responses [78,79]. GM-CSF, a cytokine modulator, promotes the differentiation of bone marrow precursors, activating and differentiating DCs, and supporting T cell homeostasis or priming of antitumor CTLs [80]. Alongside widely studied cytokines, researchers are further exploring agonists affecting immune cells through intracellular mechanisms. For instance, TGFβR1 inhibitors restore T cell activity, stimulator of interferon gene (STING) agonists promote pro-inflammatory cytokine secretion and type I interferon responses, and TLR7/TLR8 agonists directly trigger APCs to stimulate antitumor functions [81,82]. Current research explores combinations of two or more cytokines or combines cytokines with low doses of other immunotherapies in combination treatments to mitigate adverse side effects associated with high doses. Cytokine-based therapies represent a promising approach in cancer immunotherapy; however, their clinical application is limited by several challenges. High-dose cytokine administration is often associated with severe inflammatory toxicity, which can lead to serious complications such as systemic inflammation, organ failure, and shock. In addition, the inherently short half-life of most cytokines reduces their bioavailability, necessitating frequent dosing [83,84]. This not only compromises treatment efficacy but also increases the risk of adverse effects, underscoring the need for strategies to enhance cytokine stability and minimize toxicity.

**ACT** represents a highly personalized and potent T cell-based immunotherapy involving the infusion of mature T cells into the patients for targeted tumor cell elimination and prevention of cancer recurrence [85]. In metastatic tumor models, ACT includes the ex vivo isolation and expansion of TILs, followed by their reinfusion in vivo with IL-2 to generate lytic T cells for effective therapy across various tumor types [86]. Genetic modifications of T cell-based immunotherapy include CAR-T and T cell receptor (TCR) T cell therapies, where patient-derived T cells are engineered ex vivo to express receptors recognizing tumor-associated antigens (TAAs), and improving T cell-mediated tumor cell suppression [87]. CAR-T therapies, a notable form of ACT, involve genetically modifying T cells in vitro to express CARs that recognize specific antigens, subsequently reinjecting them into the patient for enhanced tumor cell death [88]. Recent advancements focus on engineering CAR-T therapies to address the immunosuppressive TME and counteract T cell exhaustion [89]. TCR-T therapies are MHC-dependent immunotherapies effective against hematological and solid tumors, recognizing TAAs like neoantigens and tumor testis antigens presented by MHCs [90]. Another ACT variant, TIL therapy, involves the ex vivo expansion of T cells harvested from a patient’s tumor for subsequent reinfusion to combat the malignancy [91]. ACT stands as a promising avenue for personalized cancer therapy. Although ACT has made significant advancements, several challenges limit its effectiveness and broader clinical application. Key obstacles include severe potentially life-threatening toxicities such as cytokine release syndrome and immune effector cell-associated neurotoxicity syndrome [87,92]. Furthermore, T cell exhaustion from prolonged in vitro expansion, antigen escape, and restricted tumor infiltration further reduces its efficacy. Practical challenges, including high manufacturing costs, long production times, and limited patient accessibility, also hinder its widespread use [73,87]. Therefore, innovative strategies are needed to enhance the potency of ACT, improve antitumor immunity, and minimize treatment-related toxicity.

**Cancer vaccines** constitute an innovative category of immunotherapies with the goal of instigating immune responses against tumor cells, falling into prophylactic and therapeutic subtypes. Prophylactic vaccines, exemplified by the human papillomavirus (HPV) vaccine, aim to prevent specific cancers by immunizing against cancer-related infectious agents [93,94]. Conversely, therapeutic cancer vaccines are designed for patients with existing malignancies, intending to enhance the immune response against tumor cells. Common types of cancer vaccines include DC vaccines, nucleic acid vaccines, tumor cell lysates (TCLs), and neoantigens. Engineered to present TAAs to the immune system, DC vaccines directly activate antitumor responses through T cells targeting tumor cells [95]. For instance, Sipuleucel-T, a personalized therapeutic vaccine for metastatic castration-resistant prostate cancer, targets the prostatic acid phosphatase [96]. Previous evidence supports the promising approach of DC-based vaccination therapy, enhancing antigen-specific T and B cell activity and CD8^+^ cytotoxic lymphocyte accumulation within the TME [97]. Understanding DCs and their role in tumor immunity, coupled with the capacity to harness TAAs, is crucial for inducing the targeted expansion of specific DC subsets with enhanced antitumor activities and creating more effective vaccines. Combining DC vaccines with other cancer immunotherapy strategies may further improve the antitumoral response [98].

**Nucleic acid-based vaccines** like DNA or RNA vaccines offer a promising alternative to conventional vaccines by delivering exogenous nucleic acids into target cells to trigger an immune response. Both DNA or RNA vaccines are taken up by APCs and transformed into antigenic proteins, stimulating immune responses against target proteins to eliminate tumor cells expressing those antigens [99]. Likewise, TCL-based vaccines contain various TAAs, which may be generated through processes like ultraviolet B ray irradiation or freeze–thaw cycles to prevent ineffective immunization due to the loss of a single antigen after cancer mutation. Clinical studies have demonstrated that some engineered tumor cells express additional immune-related molecules (interleukins or costimulatory molecules), allowing TCL vaccines to induce in vivo antitumor immune responses after administration [100]. Other than these cancer vaccines, neoantigen vaccines represent a growing area of development and are explored as cancer immunotherapies for their ability to improve immune responses against tumor cells. Neoantigens are TAAs arising from somatic DNA alterations in tumor cells, promoting antitumor immune responses [100]. Overall, cancer vaccines are rapidly evolving, with ongoing research efforts aimed at enhancing their efficacy across a broader spectrum of cancer types. Cancer vaccines offer a promising and innovative strategy for cancer treatment. However, their effectiveness is often limited by several challenges, including the complexity and variability of the TME, the presence of immunosuppressive cells, and the ability of cancer cells to evade immune detection [101]. Additionally, identifying specific tumor-associated antigens and developing personalized cancer vaccines present significant hurdles, particularly in terms of manufacturing, cost, and optimizing clinical testing [73].

**Oncolytic viruses (OVs)** represent a promising category of cancer immunotherapies with distinct advantages, including self-replication within tumor cells, induction of immunogenic cell death (ICD), and stimulation of antitumor immunity [102]. OVs not only directly eliminate tumor cells directly through viral replication but also transform “cold” tumors into “hot” tumors by modulating the release of immune-associated molecules (PAMPs, TAAs, and DAMPs). This process improves tumor immunogenicity, promotes the maturation, migration, and infiltration of immune cells, and thereby augments antitumor immune activity. Additionally, OVs impact tumor neovascularization and suppress tumor development [103]. Recent studies demonstrate that specific gene insertion into OVs, like herpesvirus type-1 (HSV-1), induces tumor antigen presentation to Teffs, leading to cancer cell elimination. Talimogene laherperepvec (T-VEC), an FDA-approved oncolytic HSV-1, produces GM-CSF and enhances local and systemic antitumor immune responses in melanoma treatment [104,105]. Some OVs, including RIGVIR, have been used to treat melanoma without genetic modification. Oncorine, a modified type 5 human adenovirus (HAdV-C5), is designed for cells with p53 defect and is employed in treating head and neck squamous cell carcinoma [106]. Clinical studies show that combining oncolytic virotherapy with ICIs produces synergistic therapeutic effects because ICIs inhibit PD-L1 induced by OVs, whereas OVs increase CD4^+^ and CD8^+^ T cell infiltration within the TME and IFN-γ production. For example, after virotherapy with canerpaturev (C-REV), antitumor activities are observed through the upregulating of PD-L1 expression on tumor cells, DCs, and macrophages [103,107]. Therefore, whether natural or genetically modified, OVs show promise as antitumor agents, specifically targeting tumor cells without harming healthy cells. Despite the therapeutic potential of OVs, several barriers limit their efficacy in cancer treatment. One of the primary challenges is the restricted penetration and distribution within solid tumors, largely due to physical barriers such as the endothelial layer, lymphatic network, and dense ECM. These structural components impede the effective delivery of OVs to target cancer cells. Furthermore, the host’s antiviral immune response poses another significant obstacle, as innate immune activation accelerates viral clearance, thereby diminishing the therapeutic impact of OVs. Overcoming these limitations is essential for optimizing OV-based cancer therapies [108,109].

**Agonist antibodies** function as costimulatory receptor agonists, specifically binding to T cell receptors’ surfaces to activate intracellular signaling pathways, thereby enhancing T cell development, effector function, and overall survival against cancer cells. The targeted T cell receptors include costimulatory receptors like CD28 and certain members of the tumor necrosis factor receptor (TNFR) family, including 41BB (CD137 or TNFRSF9), glucocorticoid-induced TNFR-related protein (GITR, also known as TNFRSF18), and OX40 (TNFRSF4), expressed on the outer surface of APCs [110]. Ligand binding to these costimulatory receptors activates intracellular signaling, stimulating T cell development and antitumor function. TNFR family members function through the JNK, NF-κB and PI3K–PKB, or AKT pathways, contributing to T cell proliferation, survival, and effector function [111]. While agonist antibodies have demonstrated efficacy in cancer therapy, several challenges must be overcome to enhance their therapeutic potential. A major limitation is their limited penetration into solid tumors, largely due to the complex and heterogeneous nature of the tumor microenvironment. Moreover, issues related to drug delivery, including the short half-life of these antibodies and the risk of severe toxicities, such as cytokine release syndrome, pose significant safety concerns [73,112]. Addressing these barriers is critical for optimizing the clinical application of agonist antibodies in cancer treatment.

## 3. Resistance Mechanisms to Immunotherapy in Cancer

**Figure 3:**
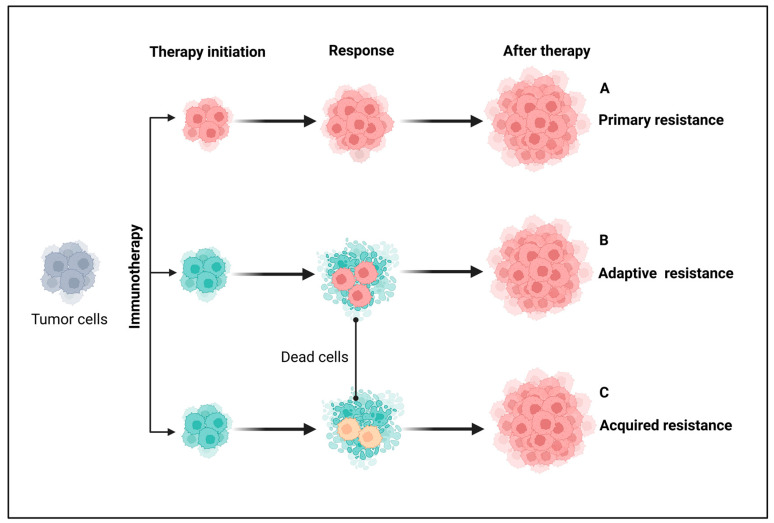
Major types of resistance to immunotherapy in patients with cancer. From a clinical perspective, immunotherapy is broadly classified into primary, adaptive, and acquired resistance based on response timing and mechanisms. (A) Primary resistance occurs when a tumor fails to respond to immunotherapy from the outset, often due to adaptive immune resistance. (B) Adaptive immune resistance arises when tumor cells evade destruction by modifying their response to immune attacks, potentially leading to primary resistance, mixed responses, or acquired resistance. (C) Acquired resistance occurs when a tumor initially responds to immunotherapy but later relapses and progresses after a period of control. Grey-colored cells indicate either non-resistant or non-sensitive tumor cells before immunotherapy. Pink- and yellow-colored cells represent different resistant tumor cells and cyan-colored cells indicate sensitive tumor cells. Created using BioRender.com.

To date, cancer immunotherapy has revolutionized cancer treatment, particularly with the development of immunomodulators like ICIs. Despite significant preclinical and clinical success, resistance to immunotherapy remains a major challenge, limiting its efficacy for many cancer patients [1,2]. The FDA-approved immunotherapies for cancer include antagonistic monoclonal antibodies that target ICs like CTLA-4 and PD-1/PD-L1, aiming to modulate the antitumor T-cell immune response. However, therapeutic resistance continues to be a key limitation in the clinical application of cancer immunotherapies, prompting a growing concern in understanding the mechanisms underlying the resistance. Broadly, clinical resistance to cancer immunotherapies can be classified as primary, adaptive, and acquired resistance based on the time of response and different mechanisms involved, as illustrated in Figure 3 [113].

**Figure 4:**
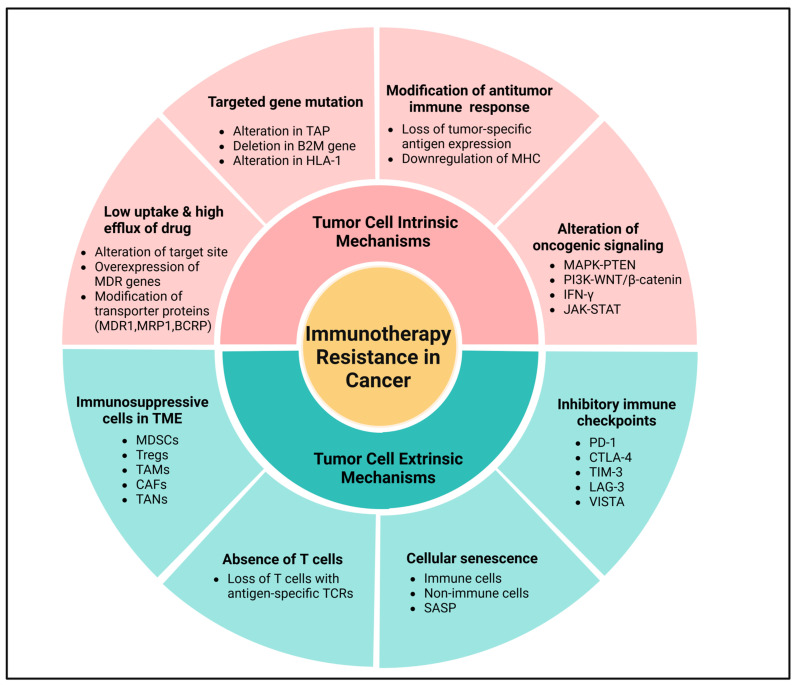
Molecular mechanisms of immunotherapeutic resistance in cancer. Tumor cell intrinsic mechanisms involve low uptake and high efflux of drugs, mutation in the targeted genes, loss of tumor-specific antigen expression through lack of T cell responses, downregulation of MHC, alteration of oncogenic signaling via MAPK pathway, and loss of PTEN expression, which promote PI3K signaling and WNT/β-catenin expression signaling pathways, and loss of IFN-γ signaling pathways. Meanwhile, tumor cell extrinsic mechanisms primarily include immunosuppressive cells in the TME, absence of T cells, cellular senescence, and expression of inhibitory immune checkpoints in the tumor cell. Created using BioRender.com.

**Figure 5:**
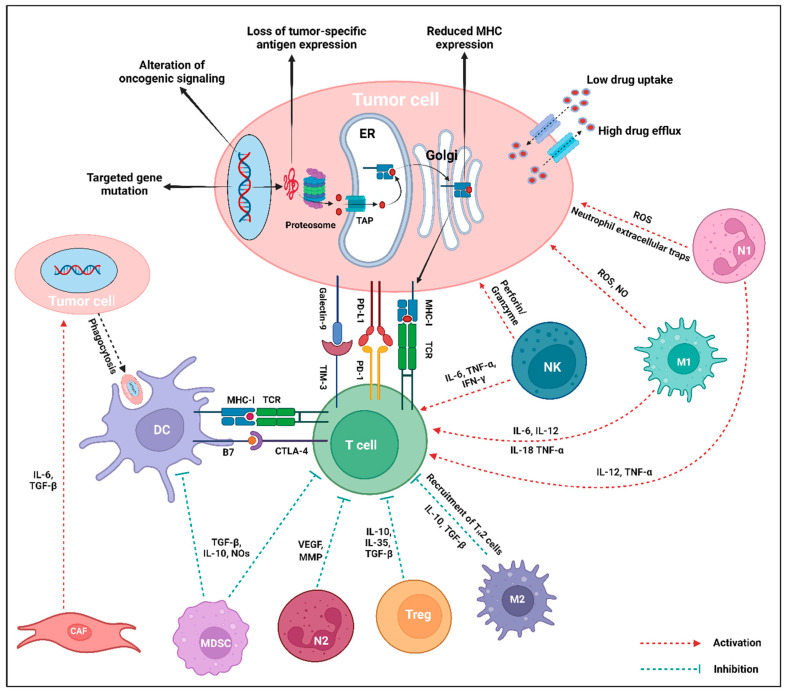
Intrinsic and extrinsic mechanisms of immunotherapy resistance in tumor cells. The central interactions between different stromal and immune cells of the TME are mediated via the release of corresponding cytokines. Within the TME, tumor-promoting cell populations like CAF, MDSC, N2-TAN, Treg, and M2-TAM either directly promote tumor growth, inhibit the antigen presentation, or directly inhibit the T cell to downregulate the immune response against the tumor cells. Tumor-suppressing cells, particularly NK, M1-TAM, and N1-TAN, enhance T cell response or exert direct tumor-killing activity. The intrinsic mechanisms of resistance in tumor cells include mutation of the targeted gene, modification of oncogenic signaling pathways, deprivation of tumor-specific antigen expression, decreased MHC expression, and low uptake and high efflux of drugs. On the contrary, tumor cell extrinsic mechanisms of resistance mostly include expression of inhibitory checkpoint molecules (PD-L1, Galectin-9 in tumor cells, or PD-1, TIM-3, CTLA-4 in T cells), loss of T cells with specific TCR, and increased activity of immunosuppressive cell populations (MDSC, Treg, M2-TAM, N2-TAN) via the generation of cytokines in the TME. Created using BioRender.com.

Primary (intrinsic) resistance refers to cases where the tumor does not respond to immunotherapy from the outset, and the lack of an objective response in tumor cells following immunotherapy may involve the mechanisms of adaptive resistance. Adaptive immune resistance is a defense mechanism in which the tumor is identified by the immune system but evades destruction by adapting to the immune attack. Due to the dynamic interaction between the tumor and the immune cells, this can clinically present as primary, mixed response, or secondary (acquired) resistance. Acquired resistance represents a clinical scenario in which a tumor initially responds to immunotherapy but later develops resistance to immunotherapies, leading to cancer recurrence [113,114]. Recognizing the complexity and heterogeneity of immunotherapeutic resistance in cancer, we have categorized intrinsic and extrinsic mechanisms in tumor cells contributing to the suppression of antitumor immune responses, providing rationales for combination treatments to overcome resistance (Figure 4 and Figure 5).

### 3.1. Tumor Cell Intrinsic Mechanisms

Tumor cell intrinsic mechanisms encompass the inherent expression or repression of specific genes and pathways in tumor cells, influencing immune cell infiltration or hindering the immunologic TME. These mechanisms may exist from the initial presentation, emphasizing the primary resistance mechanisms, or may develop later, indicative of adaptive resistance [113,115]. Currently recognized tumor-inherent mechanisms include low drug uptake and high drug efflux, targeted gene mutation, loss of tumor-specific antigen expression impairing T cell responses, downregulation of MHC, regulation of oncogenic signaling through the MAPK pathway, and loss of PTEN expression promoting PI3K signaling and WNT/β-catenin expression pathways, and loss or dual role of IFN-γ signaling pathways, as shown in Figure 4 [116].

The challenge of cancer immunotherapy is caused due to multidrug resistance gene amplification, which encodes a transmembrane protein inhibiting drug entry into the tumor cells during drug elimination [117]. Due to this alteration, a drug may reach the target site but may fail to attain the minimum effective concentration, leading to ineffective killing of the tumor cells. Notably, transporter proteins like multidrug resistance protein 1 (MDR1), multidrug resistance-associated protein 1 (MRP1), and breast cancer resistance protein (BCRP) are widely reported contributors to drug resistance in various tumors. These proteins, with specificity for eliminating foreign substances from cells, enable tumor cells to develop drug resistance [118]. The emergence of immunotherapeutic resistance diminishes tumor antigen presentation, reducing tumor recognition and destruction by the immune system, particularly following chemo-resistance. Current therapeutic strategies involve combining different therapies to counteract the mechanisms of therapeutic resistance, therefore extending the overall survival of patients with cancer [115,117]. Ultimately, the effectiveness of treatment diminishes when the drug target undergoes alterations, leading to therapeutic resistance in tumor cells.

Generally, tumor cells express neoantigens, specifically tumor-specific antigens that can serve as potential targets and beneficial diagnostic biomarkers for cancer treatment. The neoantigen burden significantly influences tumor immunogenicity, playing a crucial role in the efficacy of ICIs. Therefore, low tumor antigenicity can lead to primary resistance to immunotherapy. A diminished production of neoantigen and reduced tumor-specific antigenicity result in the loss of or decreased recognition by T cells [119]. For instance, the expression of long-stranded non-coding RNA in triple-negative breast cancer (TNBC) promotes intrinsic tumor suppressors like p53 and Rb, leading to the degradation of antigenic peptide loading systems and intrinsic tumor suppression with antigenic downregulation [120]. Some evidence indicates that tumor neoantigens contribute to improving antitumor immune responses and counteracting immunosuppressive efficacy in certain cancers [121]. Additionally, in a sarcoma mouse model, T lymphocytes targeting tumor neoantigens have demonstrated antitumor activity in response to ICIs. Consequently, the development of neoantigen-based cancer vaccines is expected to be an effective approach for combating therapeutic-resistant solid tumors [122].

Recognition of tumor antigens by CTLs occurs when the antigens engage with MHC-I and are presented on the surface of tumor cells. To evade destruction by T cells, cancer cells downregulate the surface MHC-I antigenic pathways [123]. Although IFN has been demonstrated to induce or enhance the MHC-I antigen presentation machinery, the lack of antigen presentation and processing can result in reduced MHC-I surface expression [124]. Alterations in transporter-associated proteins (TAPs) or proteasome subunits, deletion of beta-2-microglobulin (B2M) genes, and secondary changes in human leukocyte antigen-1 (HLA-1) class molecules can contribute to reduced MHC-I secretion in tumor cells, leading to resistance to tumor immunotherapy [5,125,126]. Advanced immune-targeted therapeutic strategies aimed at addressing these alterations are anticipated to maintain MHC expression on tumor surfaces, therefore enhancing the tumor-killing activity of T cells [126].

Oncogenic signaling pathways play an essential role in therapeutic resistance at various stages of tumor progression, including cancer initiation, invasion, and metastasis. For instance, the MAPK pathway in tumors produces VEGF, IL-6, and IL-10, which are known to inhibit T cell recruitment and functions. However, combining MAPK signaling inhibition with PD-1/PD-L1 treatment and/or BRAF-targeted therapies elicits antitumor immune responses, including an improved presence of TILs [127]. Likewise, therapeutic resistance to ICIs may result from the loss of PTEN expression, which activates the PI3K signaling pathway, promoting tumor cell proliferation. In melanomas, PTEN loss correlates with a reduced expression of IFN-γ, granzyme B in immune cells, and CD8^+^ T cell infiltration. Notably, PTEN mutations are more prevalent in non-T-cell-inflamed tumors, making PTEN-deficient tumors more susceptible to ACT than PTEN-expressing tumors in mouse models. Somatic PTEN mutations are also linked to therapeutic resistance in immunotherapy, potentially contributing to immunosuppression in non-responders with primary brain tumors [128]. In various cancers, the abnormal activation of the WNT/β-catenin signaling pathway in tumor cells has led to the consideration of WNT signaling inhibitors in cancer therapy. However, intrinsic WNT signaling through β-catenin stabilization may result in T cell exclusion from the tumor environment, diminishing antitumor immunity and advancing immunotherapeutic resistance. For example, cancers with raised β-catenin signaling in murine models decrease the number of CD103^+^ DCs due to reduced CCL4 expression. ICIs exhibit higher effects in targeting β-catenin-losing tumors than in β-catenin-expressing tumors, and genes associated with β-catenin signaling are more expressed in non-T-cell-inflamed tumors. In human cancers, mutations in β-catenin signaling-related genes are significantly enhanced in non-T-cell-inflamed tumors, indicating that the activation of the WNT/β-catenin signaling pathway is prevalent in about 90% of investigated non-T-cell-inflamed tumors [128,129].

IFN-γ signaling pathways are increasingly recognized as pivotal in immunotherapy resistance [130], exhibiting both positive and negative impacts on antitumor immune responses. IFN-γ generated by tumor-specific T cells contributes to potent antitumor immunity by enhancing tumor antigen presentation, recruiting diverse immune cells, and inducing antiproliferative and proapoptotic effects on the tumor cells. Conversely, tumor cells can evade detrimental immunity by downregulating or mutating molecules involved in the IFN-γ signaling pathway or controlling the immunosuppressive activities of IFN-γ [130]. For instance, T-cell responses against TAAs lead to IFN-γ expression within the TME, activating JAK-STAT signaling and the induction of PD-L1 expression. This process can be disrupted by interfering with tumor cells’ response to IFN-γ signaling pathways, rendering PD-1/PD-L1 blocking less effective [131]. Nonetheless, this strategy not only represents a resistance mechanism to immunotherapy but also to antitumor immunity. Notably, mutations in IFN-γ signaling-related genes (IFNγ-R1/R2, JAK2, and IRF1) are prevalent in non-responders to anti-CTLA-4 mAb (ipilimumab), highlighting the role of deficient IFN-γ signaling-associated genes as a resistance mechanism to anti-CTLA-4 treatment. Patients with cancer with such mutations are probably resistant to anti-PD-1 treatment due to the induction of PD-L1 expression following IFN-γ exposure [128,130]. Consequently, immunotherapies that particularly address these signaling pathways are anticipated to be a promising approach for overcoming therapeutic resistance in cancer immunotherapy (Figure 4 and Figure 5).

### 3.2. Tumor Cell Extrinsic Mechanisms

Extrinsic mechanisms within tumor cells encompass various factors such as MDSCs, Tregs, TAMs, CAFs, TANs, and ICIs, all of which may contribute to cancer immunotherapeutic resistance, as illustrated in Figure 4 and Figure 5 [132]. Tumor cells produce various cytokines and chemokines that engage all common immunosuppressive cell populations (Tregs, B cells, MDSCs, TAMs, and TANs) within the solid TME. These immune cells directly suppress the cytotoxic activity of CD8^+^ T cells and NK cells, enabling cancer cells to evade immune destruction [132]. Immunotherapy has significantly advanced cancer treatment by activating antitumor immune responses, yet its efficacy can be undermined by the expansion of Tregs, which promote an immunosuppressive TME [133,134]. Tregs, characterized by FoxP3 expression, suppress effector T cell function through cytokine secretion (IL-10, TGF-β) and metabolic disruption, ultimately dampening therapeutic responses [135]. ICIs, particularly anti-PD-1 and anti-CTLA-4, can inadvertently enhance Treg proliferation, further limiting treatment success [136]. Tumors exploit immunoregulatory pathways by recruiting Tregs, thereby fostering immune evasion and resistance [137]. For instance, Tregs are known to suppress Teffs responses through the production of suppressor cytokines like IL-10, IL-35, and TGF-β or via direct cellular intercommunication [138]. Murine models have demonstrated a significant association between an increased ratio of Teffs to Tregs ratio and anti-CTLA-4 treatment responses, particularly when based on Fcγ receptor-expressing macrophages, suggesting that utilizing anti-CTLA-4 with increased FcγR binding may promote robust antitumor immune responses and enhanced overall survival [139]. Correspondingly, MDSCs modulate the immune system through various mechanisms, including the secretion of immunosuppressive cytokines (IL-10, IL-35, TGF-β, and ROS), iNOS, Arg1 and ICI expression, and synergy with Th17 or Tregs [140]. Within TME, MDSCs are nearly related to the effectiveness of immunotherapy, suggesting that targeting tumor cells through the deactivation of macrophage PI3Kγ and ICI partnership may overcome resistance to immunotherapy [141].

Furthermore, TAMs are implicated in antitumor responses to tumor immunotherapy by expressing PD-L1 and producing IL-10, which promotes tumor development through the release of MMPs and VEGF, activating angiogenesis, altering epithelial cell motility, and recruiting Tregs or MDSCs [142]. Thus, targeting various immunosuppressive cells at different stages of tumor development may be a potential immunotherapeutic strategy to inhibit tumor immune escape. TAM recruitment is facilitated by effector proteins (CSF1 and VEGF) in tumor cells, and disrupting the CSF1/CSF1R signaling pathway is pivotal for the TAM recruitment to tumor sites, potentially synergizing with immunotherapy in refractory malignancies [142,143]. VEGF within the TME serves as a key driver of cancer neo-angiogenesis and exerts immunosuppressive functions [144]. Non-responders to anti-PD-1 therapy have exhibited higher VEGF levels than responders, suggesting a potential role of VEGF in resistance to tumor immunotherapy [145]. Additionally, chemokines play a role in the recruitment of immune cells within the TME, influencing tumor cells and ECs to modulate the proliferation of tumor cells, neo-angiogenesis, and tumor progression. Several chemokines (mainly CC, CXC, CX3C, and C) have diverse functional roles, exhibiting both pro-tumorigenic or antitumorigenic effects in different tumor models that impact immunotherapy resistance [146]. Other than the other immunosuppressive cells, CAFs have been considerably exhibited to contribute to tumor heterogeneity and intratumoral tissue heterogeneity, which correlates with clinical outcomes. For instance, the stromal microenvironment creates the intratumoral composition of pancreatic cancer, potentially linked to resistance to ICIs. However, CAFs can also immediately contribute to conditions of tumor immune desertion and rejection [147]. The expression of some genes linked to CAFs has been found to impact T cell infiltration and resistance to PD-1/PD-L1 blockade [148]. In addition, TGF-β signaling, activated in CAFs, has been correlated with resistance to PD-1 blockade in melanoma or bladder cancer [149]. Additionally, CAFs can impact resistance to ICIs through complex mechanisms involving matrisomics, secretomics, and metabolomics. Previous studies indicate that CAF-related secretome, either directly or indirectly, hampers antitumor immunity, and the quantity of CAF-derived matrix fibers firmly impacts the migration and localization of T cells [150]. The co-expression of various ICIs (PD-1, CTLA-4, LAG-3, TIM-3, and VISTA) has been demonstrated to promote the persistence of T cells, contributing to the development of resistance to ICIs [151]. Thus, monoclonal antibodies targeting multiple ICIs may serve as effective anticancer therapies.

Cellular senescence, a state of irreversible cell cycle arrest, plays a dual role in various cancers, initially acting as a tumor-suppressive mechanism but later contributing to therapeutic resistance and tumor progression. Recent studies highlight that cellular senescence in both immune and non-immune cells within the TME can hinder the efficacy of immunotherapy and drive tumor recurrence [152]. Senescence in immune cells, including T cells and macrophages, leads to immune dysfunction, reducing antitumor immunity and impairing responses to ICIs [153]. Meanwhile, senescent non-immune cells, such as fibroblasts and endothelial cells, often acquire a senescence-associated secretory phenotype (SASP), releasing inflammatory cytokines, growth factors, and extracellular matrix components that promote immune evasion and tumor progression [154,155]. Senescence immune cells, particularly T cells and DCs, can significantly alter their functionality, impeding effective antitumor immune responses by creating a suppressive TME [152,156]. This phenomenon is critical since accumulated senescent immune cells contribute to the tumor’s evasion of immune surveillance during tumor progression and post-therapy recurrence [152,156]. Thus, limiting the development of senescent T cells may amplify tumor-specific immunity and improve therapeutic outcomes [157,158]. Other than immune cells, the senescence of non-immune cells, including tumor cells themselves, plays an equally significant role in determining the fate of immunotherapy. The senescent tumor cells may become highly immunogenic by enhancing the presentation of self-antigens, potentially leading to a more robust immune response against tumors [159,160]. The accumulation of senescent immune cells can also contribute to an immunosuppressive microenvironment by increasing Tregs and MDSCs, further blunting the efficacy of immunotherapy [161]. Given these challenges, targeting senescence with senolytic drugs (which selectively eliminate senescent cells) or SASP inhibitors has emerged as a promising strategy to enhance immunotherapeutic responses and reduce tumor relapse [162]. However, this immunogenicity faces challenges under the suppressive immune microenvironment in the TME, where factors like the PD-L1 expression on senescent cells contribute to immune escape mechanisms [163,164]. This intricate interplay underscores the necessity for strategic planning to optimize the immunotherapy approaches that can selectively reduce the senescence of antitumor immune cells while simultaneously increasing senescent tumor cells to activate robust immune responses, increasing the effectiveness of immunotherapy.

## 4. Emerging Approaches and Overcoming Immunotherapeutic Resistance

With new insights into the mechanisms of resistance to tumor immunotherapy, various strategies can be adopted to combat resistance to immune-targeted drugs. A multidisciplinary strategy that incorporates advanced imaging, biomarker identification, and innovative technologies is emerging as a promising approach for robust early screening. Techniques like liquid biopsies, involving the analysis of circulating tumor DNA and circulating tumor cells, along with the identification of specific biomarkers such as epigenetic DNA modifications, exosomes, proteins, and cancer metabolites, provide valuable insights [117,165]. The integration of multi-omics and CRISPR/Cas9 gene editing technology proves beneficial in early-stage cancer lesion detection and the exploration of molecular mechanisms underlying treatment failure [166,167]. Additionally, cutting-edge computational technologies like single-cell RNA sequencing, mass cytometry, artificial intelligence (AI), and machine learning play a pivotal role in analyzing complex diagnostics, thereby facilitating biomarker identification [165,168]. Established novel biomarkers, including TMB, TIL status, PD-L1 expression, genomic markers, and the evaluation of longitudinal tumor samples throughout treatment, along with newer assessments like tumor immunograms, can be utilized to design biomarker-driven personalized treatment strategies for tumor immunotherapy. Personalized immunotherapy is transforming cancer treatment by integrating precision medicine, biomarker-based treatment strategies, and advanced technologies to enhance therapeutic efficacy while reducing severe toxicity. Utilizing molecular and genetic profiling, this approach customizes treatment for individual patients, improving response rates and minimizing adverse inflammatory effects [169]. AI and machine learning significantly contribute to predicting treatment responses by analyzing extensive datasets, as exemplified by IBM Watson for oncology and the D-CRAFT algorithm [170]. Key biomarkers such as PD-L1, TMB, and microsatellite instability (MSI) are essential in guiding ICI therapies, but emerging predictive biomarkers like neoantigen load and immune cell infiltration patterns further refine patient stratification [171,172]. Given the complexity of tumor heterogeneity, personalized treatment strategies require in-depth molecular profiling to address variations within and between tumors [173]. The integration of genomic and immunological data supports the development of novel immunotherapies, including CAR T-cell therapy and tumor-specific neoantigen vaccines, thereby improving individualized cancer care [174]. Ultimately, personalized immunotherapy combines precision medicine, biomarker-driven treatment strategies, and approaches to address tumor heterogeneity, creating customized treatments that improve efficacy and minimize toxicity. This method not only boosts the chances of successful outcomes but also advances oncology by providing more individualized care and better outcomes. Such strategies hold promises for better predictions and reducing potential instances of resistance [113,175]. Furthermore, recent studies have identified immune-related long non-coding RNAs, TAF gene signatures, heme oxygenase 1- related genes, multiple TME signatures, fecal microbiomes, and radiomic characterization all of which show potential to improve the predictability of immunotherapy effectiveness while simultaneously reducing the occurrence of irAEs in patients [13,176].

Moreover, targeting immunosuppressive cells such as Tregs can also be an effective strategy to improve the therapeutic outcomes in cancer. Treg-mediated resistance to ICB involves the upregulation of alternative checkpoint molecules such as TIM-3 and LAG-3 in Tregs, along with the release of immunosuppressive adenosine from apoptotic Tregs during PD-1 therapy [1]. Given their critical role in resistance to anti-PD-1 or PD-L1 antibodies, targeting Tregs through depletion or reprogramming could enhance the efficacy of these therapies and help overcome treatment resistance. Likewise, targeted drug delivery systems offer a promising strategy to selectively deplete Tregs in the TME, enhancing the antitumor response and improving ICB efficacy [177]. Weakening Treg dominance improves the tumor immune response, significantly boosting treatments like anti-PD-1 therapy. Additionally, emerging strategies such as Treg depletion therapies and functional modulation are being explored to enhance immunotherapy efficacy [178]. Overcoming Treg-mediated suppression is critical for improving long-term treatment outcomes and optimizing immunotherapeutic interventions. The synergistic combination of immunotherapies with conventional treatment modalities like radiation, chemotherapy, or targeted therapy can significantly enhance the antitumor potential of immune-targeted drugs and alleviate immuno-resistance. This multifaceted treatment approach has been demonstrated to induce ICD and regulate the IFN and PTEN pathways, markedly increasing tumor immunogenicity, improving the immunosuppressive TME, overcoming T-cell exhaustion, enhancing tumor T-cell infiltration, and, thereby, boosting the immune system function and transforming the immunologically “cold tumors” into “hot tumors” [113,175]. Recently, dual or triple combinations have demonstrated effectiveness in clinical trials, with some receiving FDA approval for treating multiple cancers. Beyond the inhibition of PD-1/PD-L1 and CTLA-4, the simultaneous blocking of multiple emerging co-inhibitory receptors like VISTA, TIGIT, TIM-3, and LAG-3, along with targeting stimulatory checkpoints (OX40, GITR, CD40, CD137) using bispecific antibodies, has been shown to boost the efficacy of current ICI therapies in both preclinical and clinical scenarios [179,180]. Furthermore, novel emerging strategies that utilizes STING agonist-loaded lipid nanoparticles (STING-LNP), microbubble-assisted ultrasound-guided immunotherapy of cancer (MUSIC), and immunostimulatory oligonucleotides and microbiome modulation through probiotics and fecal microbiota transplantation envision enhancing efficacy and overcoming resistance to ICIs [181,182].

## 5. Cancer Immunotherapy: Current Progress in Clinical Outcomes

Although immunotherapy offers optimistic outcomes for cancer treatment, its effectiveness is observed in only a proportion of the population, often leading to resistance and treatment failure, requiring innovative solutions. Therefore, there is growing interest in developing rational combinations of immunotherapeutics with conventional therapies to improve response and treatment outcomes, thereby reducing treatment dosage and subsequent adverse side effects. Recently, researchers have highlighted the potency of combinatorial therapy, which can be observed in multiple clinical trials. For instance, the combination of ICIs and chemotherapy has been successfully demonstrated in clinical trials to be effective in NSCLC, SCLC, and TNBC [183]. Clinical trials have demonstrated the enhanced overall survival of patients with NSCLC treated with pembrolizumab (anti-PD-L1) combined with pemetrexed and platinum chemotherapy compared with chemotherapy alone [184]. Similarly, the efficacy of combining avelumab (anti-PD-L1) with axitinib (VEGF receptor tyrosine kinase inhibitor) and pembrolizumab with axitinib for treating advanced renal cell carcinoma (RCC) surpassed that of sunitinib as the previous standard treatment, leading to the regulatory approval of both combinations [183]. It is well reported that clinical trials have confirmed the effectiveness of combining CD25 monoclonal antibodies (mAb) with tumor antigen peptide vaccines. Patients received a single dose of CD25 mAb one week before vaccination, allowing the drug to eliminate CD25^+^ Tregs before activating antitumor-specific T cells [185]. This approach effectively reduced Tregs and prolonged progression-free survival in patients with metastatic breast cancer. Correspondingly, clinical studies on anti-CTLA-4 mAb with tumor peptide vaccines demonstrated that administrating CTLA-4 monotherapy prior to vaccination enhanced the antitumor response, improving therapeutic outcomes [186]. Similarly, early clinical trials have proved the tolerability of navoximod and recommended that its combination with ICIs may enhance antitumor immune responses via counteracting Treg-mediated immunosuppression within the TME, highlighting the potential of Indoleamine 2,3-Dioxygenase 1 (IDO1)-targeted combination therapies to improve immunotherapy outcomes in advanced solid tumors [187].

Furthermore, combining pembrolizumab with conventional neoadjuvant chemotherapy in TNBC and utilizing monoclonal antibodies like trastuzumab and pertuzumab in HER2-positive breast cancer treatment have demonstrated significant effectiveness in terms of survival [188]. In metastatic mismatch repair-deficient colorectal cancer, the combination of nivolumab (anti-PD-1) and ipilimumab (anti-CTLA-4) substantially increased the overall survival rate from 31% (nivolumab alone) to 60% [189]. Additionally, combining talimogene laherparevec (a modified herpes simplex virus) with ipilimumab showed an improved response rate compared with ipilimumab alone (39% vs. 18%) in patients with advanced unresectable melanoma [190]. Similarly, the combination of pembrolizumab with body radiotherapy resulted in a twofold increase in response rate compared with ICIs alone (36% vs. 18%) for treating advanced NSCLC [191]. Nevertheless, although combination therapy is considered superior, it may elevate various toxicities. For instance, the combination of anti PD-1/PD-L1 and anti-CTLA-4 in melanoma patients led to more significant immune-related side effects [192]. Moreover, the combination of vemurafenib (a BRAF kinase inhibitor) and ipilimumab was deemed infeasible due to reported cases of hepatotoxicity [183]. In parallel, next-generation therapies like ICIs, CAR-T cells, and cancer vaccines have significantly improved survival, especially in patients with hard-to-treat cancers like NSCLC, RCC, melanoma, and hematologic malignancies. Personalized biomarker-driven approaches and innovative combinations further enhance the efficacy of immunotherapy, expanding its impact on previously resistant cancers [193]. Therefore, highlighting the importance of combination therapy for improved clinical outcomes is crucial, but it is essential to balance effectiveness with potential adverse side effects.

## 6. Conclusions and Future Directions

Exploiting the immune system has great potential for treating various cancers but achieving precise spatiotemporal control over immune function remains a major challenge that conventional cancer therapeutics struggle to address. Thus, therapeutic targeting of immunomodulation and overcoming resistance has long been regarded as a promising approach in immunotherapy armamentarium. Focusing our attention in this article, we summarized the modulation of targeted immune cells by cancer immunotherapies and their contribution to the complex mechanisms of immunotherapeutic resistance in solid tumors. We also highlighted breakthroughs in targeting immunomodulators to overcome resistance and ongoing development strategies to alleviate immunosuppressive mechanisms, aiming to activate antitumor immunity and enhance the efficacy of immune-targeted agents.

The ongoing exploration of novel immune resistance mechanisms plays a significant role in identifying fresh therapeutic targets and extending the clinical applications of immunotherapy. Nevertheless, due to the limited durable response seen in a minority of patients with ICIs, it is crucial to develop multimodal treatment approaches, including combination therapy. This approach aims to enhance clinical outcomes and address the challenge of resistance development. To address immunotherapeutic resistance and specific immune toxicity, the combination of various immunotherapy agents and the integration of immunotherapy with additional treatments like chemotherapy has demonstrated encouraging outcomes in both preclinical and clinical investigations. For instance, the enhanced response rates in patients with melanoma and RCC have been observed by dually combining anti-PD-1/PD-L1 with anti-CTLA-4 [194], and improved outcomes in patients with BRAF-mutant melanoma have been demonstrated by combining immunotherapy with targeted therapy like BRAF inhibitors [195]. Similarly, immune agonists activating specific pathways like STING and NLRP3 inflammasome pathways show potential for enhancing the antitumor immune response [196]. Furthermore, the effectiveness of immunotherapy relies on the unique characteristics of both the patient’s immune system and their individual tumor, which emphasizes the increasing focus on investigating personalized immunotherapy strategies that consider the patient’s immune status and specific tumor characteristics. For instance, current indicators like PD-L1 expression, MSI and TMB, neoantigens, and TIL status have demonstrated their efficacy as some effective predictive biomarkers for anticipating the response to ICIs [197]. Therefore, the intensive exploration of potent predictive biomarkers and the development of effective combined strategies or alternative multimodal approaches to minimize adverse toxicities are currently active areas of study representing substantial clinical challenges. Moreover, advanced 3D ex vivo models now effectively replicate the complex structure and function of the TME. These engineered tumor/organ-on-a-chip platforms offer a precise and realistic approach to studying immune suppression and Treg reprogramming therapies in cancer [198]. Furthermore, BATF may regulate Treg infiltration into human cancers by suppressing chemokine receptor signaling genes [199]. Thus, investigating BATF-expressing Tregs in combination with PD-1 blockade therapy is a promising avenue for future research.

Although comprehensive analysis, considering multiple factors and utilizing advanced technologies like whole-genome sequencing, single-cell sequencing, and epigenetic analysis, will be crucial for identifying characteristic immunotherapeutic resistance sites or sub-clones within tumors, in addition to these advanced technologies, future research should explore innovative drug delivery or microfluidics-based technologies to expand and engineer cell therapies ex vivo. This may involve the use of biomaterials or nanomaterials as carriers, contributing to the bioengineered functionalities of the platforms. While not extensively covered in this article, microbiome modulation as an adjunct therapy to ICIs that predicts clinical advantage is critical for accurately selecting patients and tailoring individualized treatments. Current discoveries suggest that the microbiome plays a role in influencing the immune response, and the enhancement of T cell-mediated antitumor immunity is achieved by microbiome modulation. However, this could pave the way for an innovative approach to boost patient response to immunotherapy, combining the regulatory capabilities of various commensal bacteria with other strategic inventions. For example, in preclinical mouse models, fecal microbiota transplantation and a *Bifidobacterium* cocktail hold promise in enhancing immunotherapy efficacy and mitigating chemotherapy-related side effects by modulating the gut microbiota [182]. Therefore, a comprehensive understanding of the complex ways in which the gut microbiota induces antitumor immunity in both preclinical and clinical settings, combined with insights into how immunotherapy impacts the gut microbiome, would advance the current knowledge and help address resistance.

## Figures and Tables

**Table 1:** Summary of the functional roles of tumor-related immune cells in solid TME.

Tumor-Related Functional Immune Cells	Cell Types	Functional Roles in Solid Tumors
**Tumor-promoting Immune Cells**	Tregs	− Releasing pro-inflammatory cytokines like IL-10 & TGF-β; − Leading to an immunosuppressive environment; − Associated with a negative prognosis.
	MDSCs	− Inhibition of T cell activity through the release of iNOS from immunosuppressive cytokines and arginine; − Linked to tumor advancement and neo-angiogenesis; − Stimulation of angiogenesis by secreting VEGF and MMP9; − Suppression of NK and T cells.
	M2-TAMs	− Promotion of tumor evasion by enhancing anti-inflammatory T_H_2 immune responses; − Production of cytokines like IL-10 and TGF-β.
	Bregs	− Releasing cytokines like IL-10 and IL-35 to inhibit the function of Teffs; − Promote an immunosuppressive cellular environment.
	N2-TANs	− Facilitation of tumor angiogenesis and metastasis through the synthesis of VEGFs, MMPs, NEs, and ROS/RNS; − Suppression of T and NK cells by eliminating tumor cells.
**Tumor-suppressing Immune Cells**	Teffs	− Inhibition of target tumor cells through granule exocytosis and initiation of Fas ligand (FasL)-mediated apoptosis; − Secretion of TNF-α and IFN-γ to enhance cytotoxicity in tumor cells.
	NK cells	− Releasing pro-inflammatory cytokines (IL-6, TNF-α, and IFN-γ) and chemokines (CCL5 and GM-CSF) to enhance antitumor immunity; − Mediating the immune response against tumors by releasing granules containing perforin and granzymes, promoting the apoptosis of tumor cells.
	DCs	− Displaying tumor antigens to naive T cells; − Releasing IL-12 and IL-18 to boost the proliferation and activation of T cells.
	M1-TAMs	− Promoting tumor rejection by generating pro-inflammatory cytokines and ROS/NO species; − Promoting vessel maturation through the secretion of anti-angiogenic cytokines.
	Beffs	− Shifting M2-TAMs towards M1-TAMs, enhancing DC maturation; − Releasing T_H_1 cytokines, promoting CTL activity, and triggering NK cell-mediated killing of tumor cells.
	N1-TANs	− Generating granules with diverse cytotoxic compounds and antimicrobials to inhibit tumor cells; − Secreting cytokines and chemokines to activate other immune cells involved in antitumor responses.

**Table 2:** Summary of key immunomodulators and their mechanisms of action in antitumor response.

Immune Modulators	Examples	Mechanism of Action	Key Effects on Antitumor Immunity	Major Limitations
Immune Checkpoint Inhibitors (ICIs)	PD-1/PD-L1 inhibitors (nivolumab, pembrolizumab), CTLA-4 inhibitors (ipilimumab), LAG-3, TIM-3, VISTA, TIGIT inhibitors	Block inhibitory immune checkpoints to restore T cell function and promote antitumor immunity	Enhanced T cell activation and tumor cell killing	Unpredictable efficacy, immune-related adverse events (irAEs)
Cytokines	IFN-α, IL-1, IL-2, IL-12, GM-CSF	Stimulate lymphocyte proliferation, macrophage and DC maturation, and inhibit tumor angiogenesis	Increased APC activation, T cell priming, and enhanced antitumor response	Inflammatory toxicity, short half-life
Adoptive Cell Transfer (ACT)	CAR-T cells, TCR-T cells, tumor-infiltrating lymphocyte (TIL) therapy	Infusion of genetically engineered or expanded T cells to enhance tumor-specific cytotoxicity	Direct tumor cell targeting and improved T cell persistence	Systemic toxicities, T cell exhaustion, high cost, and long production time
**Cancer Vaccines**	Dendritic cell (DC) vaccines, tumor cell lysates (TCLs), nucleic acid vaccines (DNA, RNA), neoantigen vaccines	Enhance antigen presentation, activate APCs, and promote tumor-specific immune responses	Increased antigen-specific T cell response and improved immunological memory	Challenges in preparing personalized cancer vaccines, manufacturing and cost barriers
**Nucleic Acid-Based Vaccines**	DNA vaccines, RNA vaccines	Delivered nucleic acids are transformed into antigenic proteins, stimulating immune responses against tumor cell antigens	Enhances immune recognition and elimination of tumor cells	
Oncolytic Viruses (OVs)	HSV-1, T-VEC, HadV-C5, C-REV	Induce immunogenic cell death (ICD), activate DCs, and stimulate pro-inflammatory responses	Enhanced APC activation, T cell infiltration, and sustained antitumor immunity	Limited delivery in tumor cells, innate immune system mediates viral clearance
Agonist Antibodies	OX40, GITR, CD137 agonists	Stimulate costimulatory pathways to enhance T cell proliferation and effector function	Improved T cell survival and expansion	Limited distribution in solid tumors, short half-life, systemic toxicities

## Data Availability

Not applicable.
